# A preoperative CT-based radiological score for predicting recurrence in papillary renal cell carcinoma: a multicenter validation study

**DOI:** 10.1186/s13244-025-02161-9

**Published:** 2025-12-08

**Authors:** Xiaoxia Li, Chenchen Dai, Jianyi Qu, Shaoting Zhang, Fan Meng, Jinglai Lin, Qi Sun, Weigen Yao, Dengqiang Lin, Ying Xiong, Jianjun Zhou

**Affiliations:** 1 Department of Radiology, Zhongshan Hospital (Xiamen), Fudan University, Xiamen, China; 2 Department of Radiology, Zhongshan Hospital, Fudan University, Shanghai, China; 3 Department of Radiology, Shandong Cancer Hospital and Institute, Shandong First Medical University and Shandong Academy of Medical Sciences, Jinan, China; 4 Department of Radiology, Affiliated Hospital of Nantong University, Nantong University, Nantong, China; 5 Department of Urology, Zhongshan Hospital (Xiamen), Fudan University, Xiamen, China; 6 Department of Pathology, Zhongshan Hospital (Xiamen), Fudan University, Xiamen, China; 7 Department of Radiology, Yuyao People’s Hospital, Ningbo, China; 8 Department of Urology, Zhongshan Hospital, Fudan University, Shanghai, China; 9 Department of Medical Imaging, Xiamen Municipal Clinical Research Center for Medical Imaging, Xiamen, China; 10 Department of Medical Imaging, Fujian Province Key Clinical Specialty for Medical Imaging, Xiamen, China; 11 Department of Imaging Big Data and Artificial Intelligence, Xiamen Key Laboratory of Clinical Transformation of Imaging Big Data and Artificial Intelligence, Xiamen, China

**Keywords:** Papillary renal cell carcinoma, Computed tomography, Prognosis, Recurrence, Scoring model

## Abstract

**Objectives:**

This study aims to establish a radiological model derived from preoperative computed tomography (CT) to predict the likelihood of papillary renal cell carcinoma (PRCC) recurrence after surgical intervention.

**Materials and methods:**

A retrospective multicenter study initially enrolled 384 patients, with 266 eligible for analysis from four centers following partial nephrectomy or radical resection for PRCC. Twelve distinct categories of CT features were evaluated. To assess reproducibility, interobserver variability in radiological assessment was evaluated. A Cox proportional hazards model was employed to identify significant radiological predictors and construct a risk score system. The model’s performance was evaluated through Harrell’s Concordance Index (C-index), and its effectiveness was compared with that of several histopathologic prognostic systems.

**Results:**

A total of 266 patients were included, comprising a training dataset from one center (*n* = 152) and an external validation dataset from three other centers (*n* = 114). Inter-reader agreement was moderate to excellent for the radiological parameters (k = 0.43–0.94). Tumor margin regularity and regional lymph node size on CT scans were found to be independently associated with tumor recurrence (subdistribution hazard ratios ranging from 5.34 to 28.11; *p*-values ranging from < 0.001 to 0.028) and were incorporated into the predictive model. The model demonstrated superior predictive accuracy for tumor recurrence in the validation set compared to existing prognostic systems (C-index: 0.95 vs. 0.74–0.92; *p*-values ranging from < 0.001 to 0.08).

**Conclusion:**

A radiological score that combines tumor margin regularity and regional lymph node size predicts PRCC recurrence, demonstrating superior performance compared to existing prognostic systems.

**Critical relevance statement:**

This CT-based scoring system outperforms existing models in prognostic accuracy, aiding clinicians in personalized risk stratification and optimizing treatment decisions for patients.

**Key Points:**

- The preoperative CT features are associated with the prognosis of papillary renal cell carcinoma (PRCC).
- Tumor irregularity and lymph node size on CT scans independently predict the postoperative recurrence of PRCC.
- A CT scoring system that incorporates these two features demonstrates superior prognostic accuracy compared to existing models.

**Graphical Abstract:**

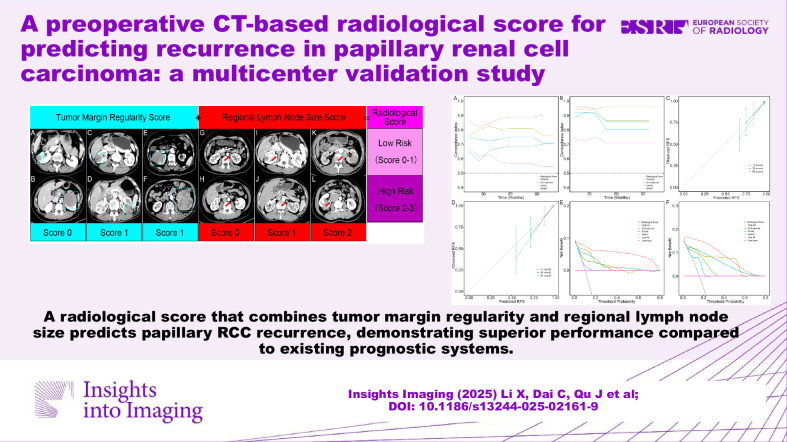

## Introduction

Papillary renal cell carcinoma (PRCC) accounts for 15–20% of renal malignancies, demonstrating disease advancement potential in roughly 10% of diagnosed cases [1]. Although the papillary subtype is associated with a favorable prognosis in renal cell carcinoma (RCC), a minority of cases exhibit a highly aggressive clinical course, leading to the poorest prognosis among the various RCC subgroups [2, 3]. A recent phase 3 study indicated that patients diagnosed with PRCC, categorized as very-high-risk, may benefit from adjuvant treatment [4]. Furthermore, individuals at higher risk of recurrence should undergo more intensive and frequent active monitoring compared to those classified as having low or moderate risk [4]. Therefore, recognizing these patients is essential for tailored clinical decisions and enhancing survival outcomes.

While several histopathologic prognostic models have been developed, many focus primarily on ccRCC [5, 6] or include all RCC subtypes [7–9], often overlooking the significant proportion of patients diagnosed with PRCC. There is a pressing need to improve prognostic models for patients with PRCC and to establish a more targeted approach for managing this second most common subtype of RCC [10]. The 2018 Leibovich prognostic model [11] and the VEnous tumor thrombus, NUclear grade, Size, T and N Stage (VENUSS) system [12] are among the limited pathological models specifically designed for PRCC. Unfortunately, evaluating pathological features often requires multiple-point assessments at critical areas, which may lead to the loss of comprehensive tumor information, and such assessments may not be available prior to surgery. Therefore, there is an urgent need to develop improved indicators that can effectively supplement current pathological staging systems to better stratify the prognosis of PRCC patients.

Emerging evidence increasingly suggests that imaging techniques, particularly contrast-enhanced CT, hold considerable promise in profiling the aggressiveness [13, 14] and prognosis of renal cell carcinoma (RCC) [15–18]. Specifically, CT-based features such as tumor contour irregularity [16, 17], the proportion of cystic components [18], heterogeneous tumor texture [15], and tumor location [19] have consistently been associated with disease outcomes. These features provide a non-invasive window into tumor biology and may serve as important prognostic biomarkers. Papillary renal cell carcinoma (PRCC) was associated with a higher likelihood of lymph node involvement [20, 21]. In our previous studies [22], we demonstrated that evaluating the CT characteristics of preoperative regional lymph nodes can yield valuable insights into disease recurrence and potential outcomes. However, whether integrating these radiological features into a comprehensive risk stratification system could improve recurrence prediction specifically for PRCC patients has not been fully addressed.

The objectives of this study were to develop and validate a prognostic risk score for predicting tumor recurrence after surgery in PRCC patients using preoperative multi-phase CT, and to compare the performance of the model with that of major histopathologic prognostic systems.

## Materials and methods

### Patients

**Fig. 1:**
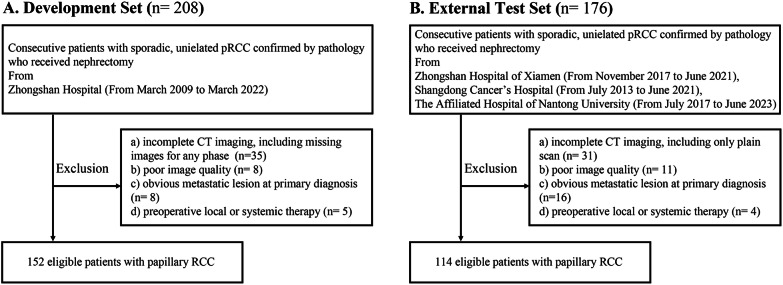
Flowchart of the inclusion and exclusion criteria of the (**A**) development set, and (**B**) external test set

This retrospective study was approved by the institutional Ethics Committee in Zhongshan Hospital Fudan University, and the committee’s reference number was B2021-608R. The requirement for written informed consent was waived. Data were collected from consecutive patients who underwent nephrectomy for sporadic cases, unilateral papillary renal cell carcinoma (PRCC) at Shanghai Zhongshan Hospital between March 2009 and March 2022 were reviewed as part of the training set. For the external test set, all included patients also had unilateral PRCC, and they underwent nephrectomy at Zhongshan Hospital of Xiamen (November 2017 to June 2021), Shandong Cancer Hospital (July 2013 to June 2021), and the Affiliated Hospital of Nantong University (July 2017 to June 2023). Patients were included if they met the following criteria: (1) they underwent contrast-enhanced computed tomography (CT) prior to surgical resection and (2) they had complete clinicopathological and follow-up data. Patients meeting any of the following criteria were excluded from the study: (1) incomplete CT imaging, including missing images for any phase, (2) poor image quality, (3) presence of obvious metastatic lesions at the time of primary diagnosis, or (4) preoperative local or systemic therapy. The inclusion and exclusion criteria are outlined in Fig. 1. The study ultimately included a total of 266 PRCC patients from the four medical centers mentioned above.

Clinical data were collected, including age, sex, Eastern Cooperative Oncology Group Performance Status (ECOG-PS), surgical procedures (such as radical or partial nephrectomy), whether lymph node dissection was performed, and details regarding adjuvant treatment. Pathological features were assessed by experienced urologic pathologists who were blinded to patient outcomes (Table 1). These features included TNM staging [23], the International Society of Urological Pathology (ISUP) grading system [24], invasion of perinephric or renal sinus fat, presence of venous thrombus, involvement of positive lymph nodes, tumor necrosis, differentiation with sarcomatoid features, and evaluation of tumor size based on the longest diameter from cross-sectional imaging. Clinicopathological variables were later utilized to categorize patients into prognostic groups based on the VEnous tumor thrombus, NUclear grade, Size, T and N Stage (VENUSS) model [12], the Stage, Size, Grade, and Necrosis (SSIGN) model [25], the 2018 Leibovich prognostic groups [11], and the GRade, Age, Nodes, and Tumor (GRANT) model [26]. Detailed scoring and grouping for these pathological prognostic models are provided in Appendix S1.

**Table 1:** Clinicopathological characteristics of PRCC patients from different cohorts

Characteristics	All patients (*N* = 266)	Development Set (*N* = 152)	External test set (*N* = 114)	*p*-value
Age^†^	58 (52–65)	58 (52–65)	57 (52–66)	0.92
Sex				0.50
Male	201 (76)	112 (74)	89 (78)	
Female	65 (24)	40 (26)	25 (22)	
ECOG-PS				**< 0.001**
0	221 (83)	115 (76)	106 (93)	
1–4	45 (17)	37 (24)	8 (7)	
Tumor size^†^	3.5 (2.5–5.3)	3.5 (2.5–4.6)	4.0 (2.5–6.2)	**0.04**
Surgical method				0.38
Radical nephrectomy	119 (45)	72 (47)	47 (41)	
Partial nephrectomy	147 (55)	80 (53)	67 (59)	
Lymphadenectomy				0.10
Yes	10 (4)	3 (2)	7 (6)	
No	256 (96)	149 (98)	107 (94)	
Adjuvant treatment				0.78
Yes	14 (5)	7 (5)	7 (6)	
No	252 (95)	145 (95)	107 (94)	
TNM stage				0.28
I	218 (82)	129 (85)	89 (78)	
II	17 (6)	7 (5)	10 (9)	
III	31 (12)	16 (10)	15 (13)	
Lymph node involvement				**0.02**
Absent	256 (96)	150 (99)	106 (93)	
Present	10 (4)	2 (1)	8 (7)	
ISUP Grade				0.54
Grade 1	13 (5)	5 (3)	8 (7)	
Grade 2	187 (70)	110 (72)	77 (67)	
Grade 3	62 (23)	35 (23)	27 (24)	
Grade 4	4 (2)	2 (2)	2 (2)	
Sarcomatoid differentiation				0.51
Absent	264 (99)	150 (99)	114 (100)	
Present	2 (1)	2 (1)	0 (0)	
Necrosis				0.16
Absent	254 (95)	148 (97)	106 (93)	
Present	12 (5)	4 (3)	8 (7)	
Perinephric or renal sinus fat invasion				0.24
Absent	241 (91)	141 (93)	100 (88)	
Present	25 (9)	11 (7)	14 (12)	
Tumor thrombus				0.75
Absent	256 (96)	147 (97)	109 (96)	
Present	10 (4)	5 (3)	5 (4)	
VENUSS group				0.26
Low risk	200 (75)	120 (79)	80 (70)	
Intermediate risk	43 (16)	21 (14)	22 (19)	
High risk	23 (9)	11 (7)	12 (11)	
2018 Leibovich group				0.77
Low risk	189 (71)	110 (72)	79 (69)	
Intermediate risk	44 (17)	25 (17)	19 (17)	
High risk	33 (12)	17 (11)	16 (14)	
GRANT group				0.67
Low risk	227 (85)	128 (84)	99 (87)	
High risk	39 (15)	24 (16)	15 (13)	
SSING group				**0.03**
Low risk	207 (78)	124 (82)	83 (73)	
Intermediate risk	46 (17)	25 (16)	21 (18)	
High risk	13 (5)	3 (2)	10 (9)	

*p*-values were computed by comparing the development and external test sets. Differences were compared with the Student’s *t*-test or Mann‒Whitney U test for continuous variables and the χ^2^ test or Fisher’s exact test for categorical variables, as appropriate. Bold values indicated statistical significance (*p* < 0.05).

*ECOG-PS* Eastern Cooperative Oncology Group Performance Status, *TNM* tumor node metastasis, *ISUP* International Society of Urological Pathology, *VENUSS* venous tumor thrombus, nuclear grade, size, T and N stage, *GRANT* grade, age, nodes, and tumor, *SSING* stage, size, grade and necrosis

^†^ Data are medians; data in parentheses are IQRs

### CT acquisition

The patients underwent preoperative contrast-enhanced CT examinations, including the pre-contrast phase (PCP), corticomedullary phase (CMP), and nephrographic phase for analysis. The detailed parameters of the CT examination are provided in Appendix S2 and Table S1.

### Imaging analysis

Three urological radiologists (X.L., C.D., and S.H., with 8, 9, and 5 years of experience in urological imaging diagnosis, respectively) independently reviewed the CT scans. Although they were aware of the PRCC diagnosis in all patients, they remained unaware of other clinicopathological data and follow-up details. Disagreements regarding the interpretation of the included features were resolved through consultation with a senior urology radiologist (J.Z., who has 30 years of experience in urological imaging diagnosis).

**Fig. 2:**
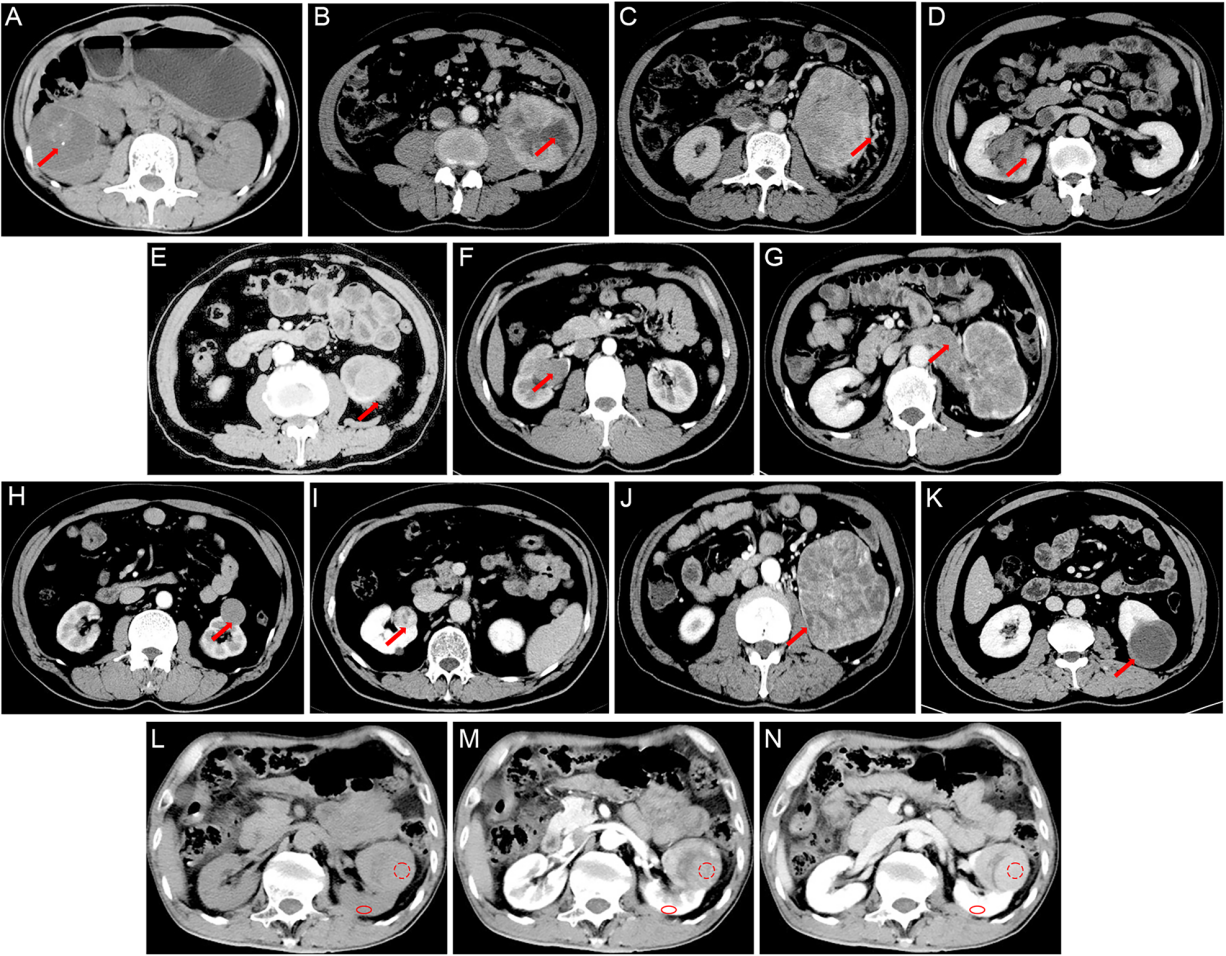
Display of image features. **A** Calcification, **B** CT necrosis, **C** peritumoral neovascularity, **D** CT renal sinus fat invasion, **E** CT perinephric fat invasion, **F** CT collecting system invasion, **G** CT vein invasion, **H** homogeneous enhancement, **I** solid or predominantly solid lesions with small areas of low attenuation, **J** lesions with mixed solid and low-attenuation areas, **K** predominantly low-attenuation lesions with peripheral enhancement. **L**–**N** The placement method of ROIs for measuring TEV and CEV. TEV, tumor enhancement value; CEV, cortex enhancement value

**Fig. 3:**
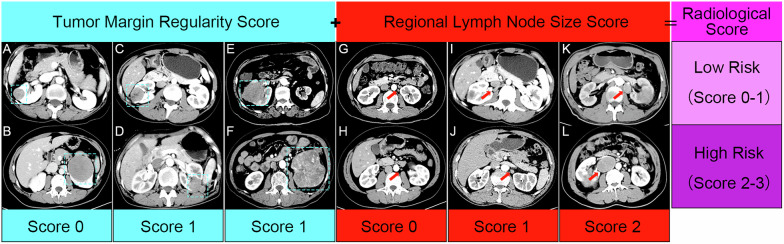
Illustration of the radiological recurrence risk scoring system (**A**–**L**)

**Fig. 4:**
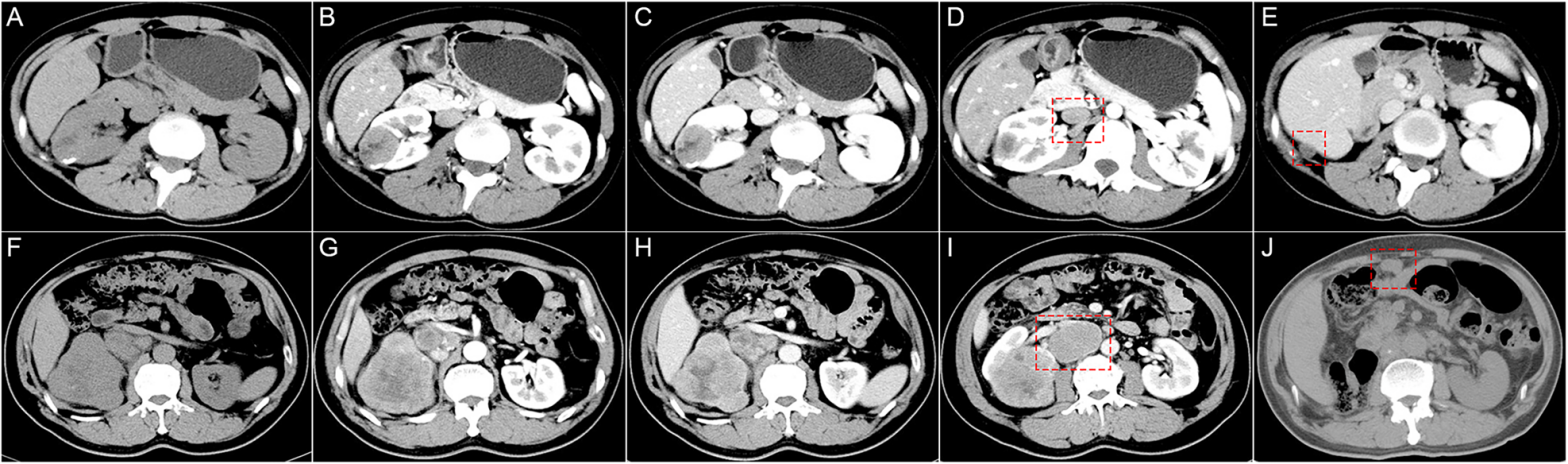
Preoperative axial contrast-enhanced CT images, from left to right: pre-contrast phase, corticomedullary phase, nephrographic phase, regional lymph node display, and post-surgery CT follow-up. **A**–**E** A 23-year-old female patient diagnosed with papillary renal cell carcinoma (PRCC) underwent radical nephrectomy. The tumor measured approximately 3.4 cm, with a tumor margin regularity score of 1 point. The short diameter of the right renal hilar lymph node was 8 mm, corresponding to a regional lymph node score of 1 point. The patient was classified as high-risk (Radiological score = 2). Subcapsular liver nodules were identified on follow-up CT 17.5 months post-surgery, which were subsequently confirmed as a recurrence of PRCC through surgical pathology. **F**–**J** A 58-year-old male patient diagnosed with PRCC underwent radical nephrectomy and lymph node dissection. The tumor measured approximately 9 cm, with a tumor margin regularity score of 1 point. The short diameter of the right renal hilar lymph node was 38 mm, corresponding to a regional lymph node score of 2 points. The patient was classified as high-risk (Radiological score = 3). Intra-abdominal nodule was identified on follow-up CT 3 months post-surgery, which was subsequently confirmed as a recurrence of PRCC through surgical pathology

**Fig. 5:**
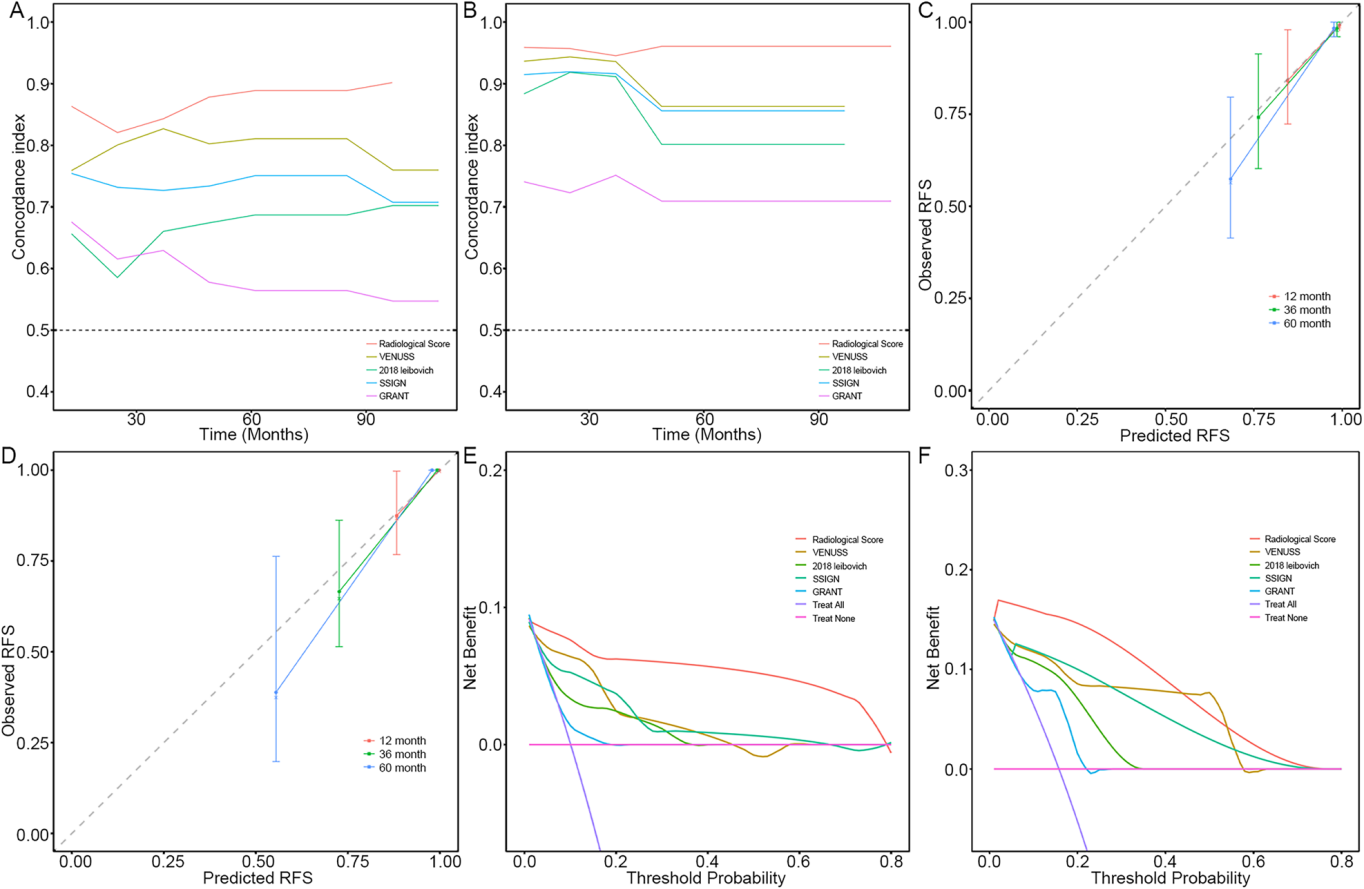
Graphs show discriminative performances. The concordance index in the (**A**) development and (**B**) external test sets. The calibration curves of the radiological score in the (**C**) development and (**D**) external test sets. Decision curves are plotted for time to recurrence at 5 years in the (**E**) development and (**F**) external test sets. VENUSS, venous tumor thrombus, nuclear grade, size, T and N stage; SSIGN, the stage; size, grade and necrosis; GRANT grade, age, nodes, and tumor

**Fig. 6:**
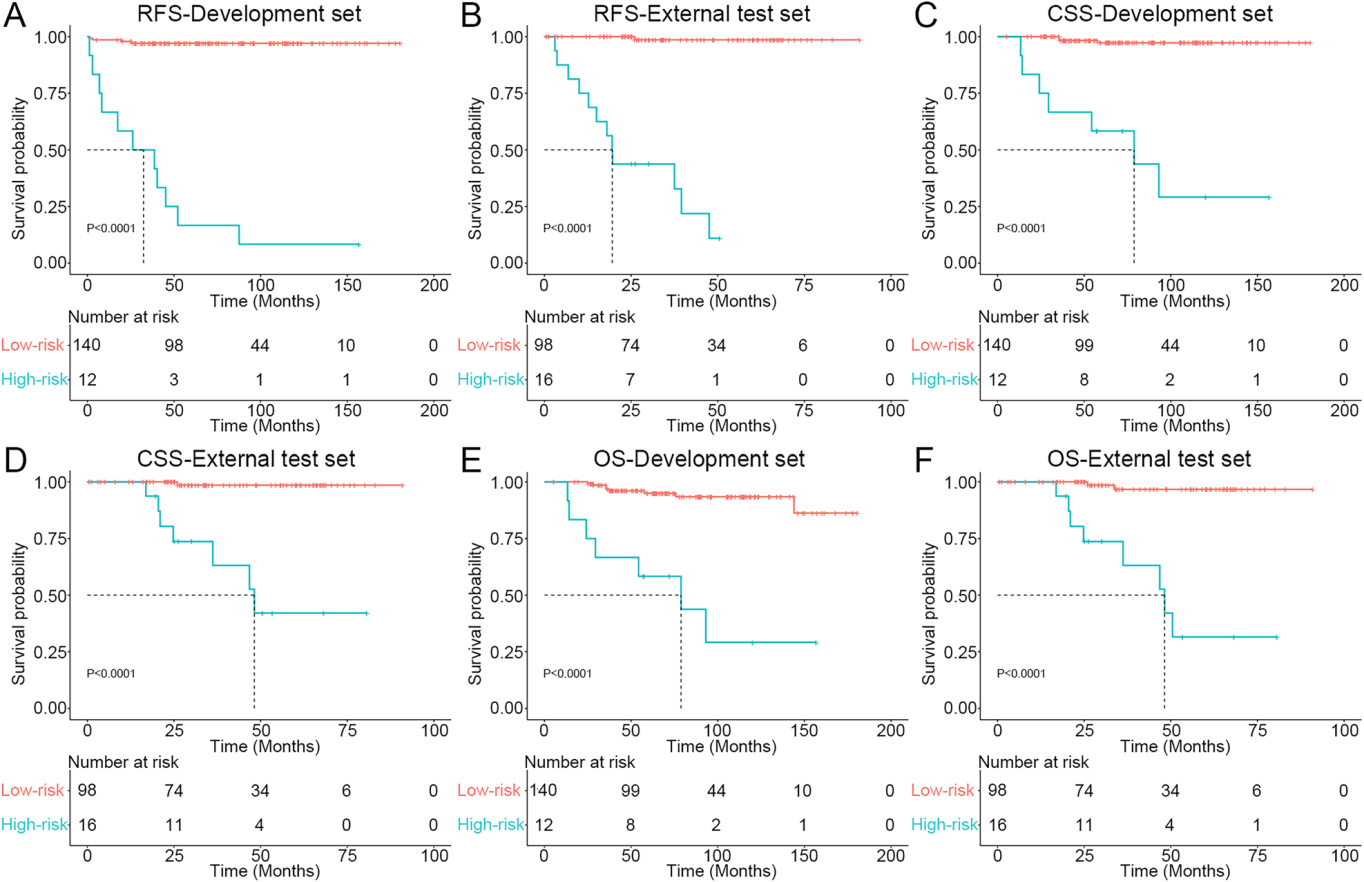
Graphs show recurrence-free survival in the (**A**) development and (**B**) external test sets, cancer-specific survival in the (**C**) development and (**D**) external test sets and overall survival in the (**E**) development and (**F**) external test set in patients with PRCC

Three radiologists independently assessed all images, evaluating the following twelve distinct categories of CT features: (1) the R.E.N.A.L. nephrometry score; (2) the presence of calcifications within the tumor (Fig. 2A); (3) the presence of tumor necrosis (Fig. 2B); (4) the presence of peritumoral neovascularity (Fig. 2C); (5) CT vein invasion (Fig. 2G); (6) CT collecting system invasion (Fig. 2F); (7) CT renal sinus fat invasion (Fig. 2D); (8) CT perinephric fat invasion (Fig. 2E); (9) tumor margin regularity (TMR), classified into three categories: TMR 1-completely regular (Fig. 3A, B), TMR 2-Irregular, with less than 50% of the entire circumference affected, including local micro-protrusions (Fig. 3C) or obvious local protrusions (Fig. 3D), and TMR 3-widely irregular, with 50% or more of the entire circumference affected, including widespread micro-protrusions (Fig. 3E) or obvious widespread protrusions (Fig. 3F); (10) pattern of enhancement, classified into four types: homogeneous enhancement (Fig. 2H), solid or predominantly solid lesions with small areas of low attenuation (Fig. 2I), lesions with mixed solid and low-attenuation areas (Fig. 2J) and predominantly low-attenuation lesions with peripheral enhancement (Fig. 2K); (11) regional lymph node size (LNS), classified using a three-point scale: LNS 1—no enlarged lymph nodes or a short-axis diameter < 7 mm (Fig. 3G, H), LNS 2—slightly enlarged lymph nodes, with a short-axis diameter ≥ 7 mm but < 10 mm (Fig. 3I, J), and LNS 3—significantly enlarged lymph nodes, with a short-axis diameter ≥ 10 mm (Fig. 3K, L); (12) degree of enhancement (Fig. 2L–N). Detailed explanations and category/score of these imaging features are provided in Table S2.

### Patient follow‑up

The main outcome measure of this study was disease recurrence, which was defined as local recurrence on the ipsilateral side, contralateral recurrence, or distant metastasis. Recurrence was determined based on follow-up CT or MR imaging evaluations and clinical records [27]. The follow-up strategy is detailed in Appendix S3. Recurrence-free survival (RFS) was defined as the time from surgery to first renal cell carcinoma recurrence (local disease or distant metastases identified by imaging, biopsy, or physical examination). The secondary endpoints were overall survival and cancer-specific survival (CSS), defined as the time from surgery to all-cause death or death from PRCC. Dates of recurrence or death were obtained from medical records or through phone inquiries conducted by an author who was not involved in the imaging analysis.

### Statistical analysis

The sample size was sufficient to ensure at least five outcome events per variable for multivariable Cox regression [28]. Intraobserver agreement was assessed using Fleiss’ kappa and the intraclass correlation coefficient (ICC). Detailed information can be found in Appendix S4.

Through the use of univariable and multivariable proportional hazards models, predictive variables were determined by applying backward stepwise selection, which was directed by the Akaike information criterion. Pearson’s correlation and variance inflation factor were used to assess multicollinearity (Appendix S4). A simple risk score was then developed using the regression coefficients of the predictor variables in the multivariate Cox proportional hazards model. The resulting score is a weighted sum of these predictors, with the weights defined as the rounded integer of the regression coefficient divided by that of the smallest reference predictor. The risk score was validated in an external test set using the same calculator. The evaluation of model discrimination utilized the concordance index (C-index) and the time-dependent area under the receiver operating characteristic curve (AUC). Calibration curves were employed to compare true survival outcomes with the predictions made by the model. To assess the clinical utility of the models, decision curves were constructed by calculating net benefits across various threshold probabilities. The model’s performance was subsequently compared with that of established prognostic systems.

Patients were categorized into high-risk and low-risk groups based on the radiological score that predicted tumor recurrence. Kaplan-Meier survival curves were then generated for each risk group and subgroup, with differences between groups assessed using the log-rank test.

Statistical analysis was carried out utilizing SPSS v.20.0 and R software (version 4.2.0). A significance level of *p* < 0.05 was applied.

## Results

### Patient characteristics

Table 1 provides a summary of the baseline characteristics of the patients. After applying the inclusion and exclusion criteria, a total of 266 patients with pathologically confirmed PRCC were retrospectively included in the study, out of an initial 384 patients (Fig. 1). The patients were divided into a development cohort from Zhongshan Hospital (*n* = 152; median age [interquartile range, IQR], 58 [52, 65] years; 112 men) and an external validation cohort from three other centers (*n* = 114; median age [IQR], 57 [52, 66] years; 89 men). Specifically, patients in the external validation cohort had a larger tumor size than the development set (median 4.0 [2.5–6.2] cm vs. 3.5 [2.5–4.6] cm, *p* = 0.04) and a higher rate of lymph node involvement (8 [7%] vs. 2 [1%], p = 0.02). The median follow-up duration was 51.7 months (IQR: 27.5–77). Among all enrolled patients, 28 (11%) experienced tumor recurrence. The recurrence sites and distant metastases identified included the liver and/or abdominal cavity (*n* = 11), lung (*n* = 12), lymph nodes (*n* = 12), (*n* = 4), bone (*n* = 6), and brain (*n* = 1). Additionally, local recurrence (*n* = 2) was observed in the ipsilateral region.

### Development of the radiological recurrence risk models

In the development set, a total of 8 variables were chosen based on the univariable analyses (*p*-value range, < 0.001 to 0.04; Tables 2, S3). Among these variables, there were no significant correlations or multicollinearity issues among the selected 8 variables (Supplementary Fig. 1, Table S4). Following multivariable analyses, tumor margin regularity (TMR) (subdistribution hazard ratio, 5.3; *p* = 0.028 for TMR 2; hazard ratio, 9.8; *p* = 0.003 for TMR 3) and regional lymph node size (LNS) (subdistribution hazard ratio, 5.5; *p* = 0.026 for LNS 2; hazard ratio, 28.1; *p* < 0.001 for LNS 3) were found to be associated with time to tumor recurrence. Subsequently, a simple radiological risk score was developed using the significant predictors identified in the multivariable model (Tables 2, 3). The radiological scores for these predictors were converted into integer values by rounding the quotient obtained by dividing each regression coefficient by the minimum regression coefficient (TMR 2), which was assigned a risk score of 1. The radiological score was calculated as follows: 1 (TMR 2: irregular, less than 50% of the entire circumference) + 1 (TMR 3: widely irregular, greater than or equal to 50% of the entire circumference) + 1 (LNS 2: ≥ 7 mm but < 10 mm) + 2 (LNS 3: ≥ 10 mm), with a score of 0 otherwise. Patients with scores of 0 to 1 were classified as low risk, while those with scores of 2 to 3 were considered high risk (Fig. 3). In Fig. 4, both the preoperative and follow-up CT images of patients with a high risk of recurrence are displayed. Inter-reader agreement was excellent for TMR, LNS, and radiological score/risk group at CT (k = 0.85–0.87) (Table 4) and moderate to excellent for the remaining parameters (k = 0.43–0.94) (Table S5).

**Table 2:** Univariable and multivariable Cox proportional hazard analyses for postoperative recurrence-free survival in the development set

Parameter	Univariable Cox proportional analysis			Multivariable Cox proportional analysis		
	Regression coefficient	Hazard ratio	*p*-value	Regression coefficient	Hazard ratio	*p*-value
**Location relative to the polar line**						
Entirely above or below the polar line	Ref.	Ref.	Ref.	…	…	…
Cross the polar line	0.87	2.4 (0.73–7.86)	0.15	…	…	…
> 50% cross the polar line crosses the axial renal midline or entirely between the polar lines	1.36	3.88 (1.09–13.77)	0.04	…	…	…
**Calcification**	1.77	5.86 (2.0–17.17)	0.001	…	…	…
**Tumor margin regularity (TMR)**						
TMR 1: completely regular	Ref.	Ref.	Ref.	Ref.	Ref.	Ref.
TMR 2: irregular and less than 50% of the entire circumference	2.73	15.34 (4.12–57.17)	< 0.001	1.68	5.34 (1.20–23.77)	0.028
TMR 3: widely irregular, greater than or equal to 50% of the entire circumference	3.31	27.34 (7.69–97.23)	< 0.001	2.28	9.79 (2.17–44.23)	0.003
**Pattern of enhancement**						
Homogeneous enhancement	Ref.	Ref.	Ref.	…	…	…
Solid or predominantly solid lesions with small areas of low attenuation	0.74	2.09 (0.3–14.88)	0.46	…	…	…
Lesions with mixed solid and low-attenuation areas	2.79	16.22 (3.44–76.45)	< 0.001	…	…	…
Predominantly low-attenuation lesions with peripheral enhancement	1.55	4.7 (0.78–28.13)	0.09	…	…	…
**Peritumoral neovascularity**	2.76	15.78 (5.3–47.01)	< 0.001	…	…	…
**CT collecting system invasion**	1.83	6.26 (2.13–18.33)	0.001	…	…	…
**CT renal vein invasion**	2.71	15.01 (4.18–53.98)	< 0.001	…	…	…
**Regional lymph node size (LNS)**						
LNS 1: No lymph nodes or a short-axis diameter < 7 mm	Ref.	Ref.	Ref.	Ref.	Ref.	Ref.
LNS 2: a short-axis diameter ≥ 7 mm but < 10 mm	2.76	15.82 (4.43–56.44)	< 0.001	1.7	5.48 (1.23–24.44)	0.026
LNS 3: a short-axis diameter ≥ 10 mm	4.23	68.83 (17.97–263.72)	< 0.001	3.34	28.11 (6.07–130.29)	< 0.001

Data in parentheses are 95% CIs

**Table 3:** Risk score for factors associated with recurrence-free survival of papillary renal cell carcinoma

Parameter	Score
Tumor margin regularity (TMR)	
TMR 1: completely regular	0
TMR 2: irregular and less than 50% of the entire circumference	1
TMR 3: widely irregular, greater than or equal to 50% of the entire circumference	1
Regional lymph node size (LNS)	
LNS 1: No lymph nodes or a short-axis diameter < 7 mm	0
LNS 2: a short-axis diameter ≥ 7 mm but < 10 mm	1
LNS 3: a short-axis diameter ≥ 10 mm	2

Risk score for each parameter was calculated based on the regression coefficient obtained from multivariable Cox proportional hazard analysis. TMR 2 was defined as a risk score of 1. The risk scores for the other parameters were the rounded quotient of the regression coefficients for the parameters divided by the regression coefficient

**Table 4:** Interobserver agreement of CT features in PRCC

Imaging features	Frequency			Kappa value	Agreement
	R1	R2	R3		
Tumor margin regularity (TMR)					
TMR 1: completely regular	225 (84)	215 (81)	224 (84)	0.86	Excellent
TMR 2: irregular and less than 50% of the entire circumference	23 (9)	34 (13)	26 (10)		
TMR 3: widely irregular, greater than or equal to 50% of the entire circumference	18 (7)	17 (6)	16 (6)		
Regional lymph node size (LNS) (mm)					
LNS 1: No lymph nodes or a short-axis diameter < 7 mm	219 (82)	224 (84)	221 (83)	0.87	Excellent
LNS 2: a short-axis diameter ≥ 7 mm but < 10 mm	33 (12)	25 (9)	31 (12)		
LNS 3: a short-axis diameter ≥ 10 mm	14 (6)	17 (7)	14 (5)		
Radiological score					
0	202 (76)	197 (74)	201 (76)	0.85	Excellent
1	36 (14)	40 (15)	38 (14)		
2	19 (7)	17 (6)	18 (7)		
3	9 (3)	12 (5)	9 (3)		
Radiological score-based risk group					
Low-risk	238 (90)	237 (89)	238 (90)	0.88	Excellent
High-risk	28 (10)	29 (11)	27 (10)		

Data in parentheses are percentages. R1, R2, and R3 were reviewers with 7, 10, and 5 years of experience in urological imaging, respectively. Agreement was considered poor (κ or ICC < 0.2), fair (κ or ICC: 0.2–0.4), moderate (κ or ICC: 0.4–0.6), substantial (κ or ICC: 0.6–0.8), or excellent (κ or ICC > 0.8). Interobserver agreement was investigated by computing the Fleiss’κ value for ordinal/categorical variables

### Validation of the radiological score

After adjusting for clinical and pathological variables (age, sex, ECOG-PS, surgical method, adjuvant treatment, pathological necrosis, pathological sarcomatoid differentiation, ISUP grade, and TNM stage), the preoperative radiological score remained an independent prognostic factor for predicting recurrence-free survival (RFS) in both sets (all *p* ≤ 0.001, Supplementary Tables S6, S7).

Figure 5 illustrates the time-dependent AUC and calibration curves at various time points. The C-indexes for the radiological score were 0.88 (95% CI: 0.77, 0.98) for the training set and 0.95 (95% CI: 0.93, 0.98) for the test set (Table 5). These results, along with the calibration curves, highlight the excellent predictive performance of the radiological score.

**Table 5:** Comparison of different model groups in predicting RFS

	Model	1-year	3-year	5-year	C-index	*p*-value^*^
Development set	Radiological score	0.87 (0.70–1.04)	0.85 (0.72–0.99)	0.91 (0.81–1.01)	0.88 (0.77–0.98)	Ref.
	VENUSS group	0.77 (0.56–0.98)	0.85 (0.71–0.98)	0.82 (0.7–0.94)	0.80 (0.69–0.92)	0.23
	2018 Leibovich group	0.66 (0.43–0.90)	0.67 (0.49–0.85)	0.68 (0.53–0.83)	0.68 (0.54–0.83)	0.006
	SSIGN group	0.76 (0.56–0.96)	0.74 (0.58–0.90)	0.76 (0.62–0.89)	0.74 (0.61–0.86)	0.05
	GRANT group	0.68 (0.48–0.88)	0.64 (0.48–0.79)	0.55 (0.43–0.67)	0.58 (0.46–0.70)	< 0.001
External test set	Radiological score	0.97 (0.94–1.00)	0.96 (0.91–1.00)	0.99 (0.98–1.01)	0.95 (0.93–0.98)	Ref.
	VENUSS group	0.97 (0.95–1.00)	0.94 (0.89–0.99)	0.87 (0.72–1.02)	0.92 (0.87–0.97)	0.08
	2018 Leibovich group	0.87 (0.78–0.95)	0.96 (0.91–1.00)	0.84 (0.68–1.00)	0.88 (0.82–0.94)	< 0.001
	SSIGN group	0.90 (0.83–0.98)	0.94 (0.89–0.99)	0.89 (0.75–1.03)	0.90 (0.86–0.95)	0.08
	GRANT group	0.82 (0.61–1.04)	0.77 (0.62–0.93)	0.73 (0.58–0.88)	0.74 (0.60–0.87)	< 0.001

Data in parentheses are the 95% CIs

*VENUSS* venous tumor thrombus, nuclear grade, size, T and N stage, *GRANT* grade, age, nodes, and tumor, *SSING* stage, size, grade and necrosis

* *p*-values were computed by comparing with the radiological score

### Survival outcomes based on radiological score

According to the radiological score, the high-risk group exhibited lower recurrence-free survival (RFS) (both sets, *p* < 0.001), lower overall survival (both sets, *p* < 0.001), and lower cancer-specific survival (CSS) (both sets, *p* < 0.001) compared to the low-risk group at different time points (Fig. 6, Table S8).

### Comparisons with existing prognostic models

The C-index of the radiological score in the test set (C-index, 0.95) was higher than that of the 2018 Leibovich group (C-index, 0.88; *p* < 0.001) and the GRANT group (C-index, 0.74; *p* < 0.001). No significant difference was observed when compared with the VENUSS group (C-index, 0.92; *p* = 0.08) and the SSIGN group (C-index, 0.90; *p* = 0.08) (Table 5, Fig. 5). The analysis of the decision curve demonstrated that the radiological score offers greater net benefits compared to the current prognostic systems at risk thresholds of 80% or lower in the training set, and 40% or lower in the test set (Fig. 5).

### Stratified analysis based on radiological score

Kaplan-Meier survival analyses demonstrated that the preoperative radiological score effectively stratified patients into prognostically distinct high-risk and low-risk groups, even within the low and intermediate-high categories defined by various models (VENUSS, Leibovich, SSIGN, and GRANT) among all 266 patients with PRCC (*p* < 0.001 for all comparisons; Supplementary Fig. 2). Notably, our results showed that recurrence-free survival (RFS) for patients classified as VENUSS low-risk PRCC in the radiological-score-defined high-risk category was significantly shorter than for patients classified as VENUSS intermediate-high risk PRCC in the radiological low-risk category. Similarly, RFS was significantly worse for patients with low-risk PRCC according to the Leibovich, SSIGN, and GRANT models (*p* < 0.05 for all comparisons; Supplementary Fig. 3).

Additionally, when stratified by tumor size, significant differences in recurrence-free survival were noted between the two risk groups, as defined by the score, in patients with tumors smaller than 3 cm (Supplementary Fig. 2). Our risk score exhibited relatively high performance in assessing the risk of recurrence in small PRCC, surpassing existing pathological models (Supplementary Table S9).

## Discussion

Accurate identification of patients with papillary renal cell carcinoma (PRCC) who are at high risk of postoperative recurrence is critical for guiding personalized treatment and improving long-term outcomes. However, comprehensive preoperative prognostic models are still underdeveloped. To address this gap, we developed a radiological scoring system that integrates CT-based features to predict the risk of postoperative recurrence. In a multicenter cohort of 266 patients undergoing either partial or radical nephrectomy, our radiological score—which comprises tumor margin irregularity (TMR) and regional lymph node size (LNS)—demonstrated a concordance index (C-index) of 0.95 in the external test set, outperforming several established clinicopathological models (C-index range: 0.74–0.92; *p*-values < 0.001–0.08).

While pathological assessment remains the gold standard for prognosis, our decision to construct a purely imaging-based model is based on both practical and biological considerations. First, pathological evaluation typically involves sampling selected tumor regions, which may not fully capture the tumor’s heterogeneity or biological aggressiveness. Second, pathological data are often unavailable preoperatively and are frequently influenced by variable surgical practices, such as lymph node dissection (LND), which may be inconsistently performed or omitted due to the lack of standardized guidelines [29]. To address these limitations, we have designed a non-invasive, preoperative imaging-based model intended to complement existing pathological systems and enhance risk stratification prior to surgery.

Why does our radiological scoring system demonstrate superior prognostic performance compared to existing pathology-based systems? This can be largely attributed to two key imaging features it incorporates: TMR and LNS. Among these, LNS is particularly noteworthy. Previous research [30, 31], including our own [22], has identified the radiologic lymph node score as an independent prognostic factor in PRCC, regardless of pathological nodal status. This discrepancy likely arises from the underestimation of nodal involvement by pathology [29], which is often due to limited or absent lymph node dissection (LND) and the lack of consistent dissection templates. Indeed, studies have indicated that patients with radiologically enlarged but pathologically negative nodes (cN1pN0) exhibit outcomes comparable to those with radiologically negative but pathologically positive nodes (cN0pN1), thereby underscoring the prognostic significance of imaging-detected lymphadenopathy [30, 31]. We adopted a 7 mm short-axis cutoff for LNS based on the work of Gershman et al, who reported that this threshold corresponds to a 20% predicted risk of lymph node metastasis [32]. Our results validated this cutoff: nodes measuring 7–10 mm were significantly associated with recurrence (HR = 5.48), while nodes greater than 10 mm conferred a markedly elevated risk (HR = 28.11), designating them as part of the high-risk group. These findings advocate for a refinement in the radiologic definition of clinically suspicious lymph nodes in PRCC, potentially lowering the conventional 10 mm threshold to 7 mm. Such a revision may provide practical guidance for surgical planning and decisions regarding lymph node dissection.

The second critical feature, tumor margin irregularity, also demonstrated strong predictive value, consistent with prior studies that link irregular margins to adverse pathology and invasive growth patterns [16, 17]. Irregular margins may reflect disrupted tumor architecture and infiltrative behavior, serving as an indirect marker of biological aggressiveness [33]. Although additional radiological features were initially considered, they were excluded from the final model, thereby reinforcing the strong standalone predictive value of TMR and LNS.

Although tumor recurrence is influenced by multiple factors, including patient comorbidities, surgical approaches, and adjuvant treatments, our radiological score remained an independent predictor of recurrence after multivariable adjustment for key clinical confounders. Importantly, the imaging score provided incremental value to several established pathological models. For instance, patients classified as low-risk by the VENUSS model but high-risk according to our imaging score experienced significantly worse outcomes than those categorized as intermediate-to-high risk by VENUSS yet low-risk by imaging. Similar trends were observed with other scoring systems. This finding suggests that our radiological score can refine risk stratification, helping to avoid overtreatment in truly low-risk patients and enabling early intervention in high-risk individuals who might otherwise be overlooked. We note that some of these stratified subgroups involve relatively small patient numbers (Table S10), which may limit statistical power; therefore, these findings need to be validated in larger subgroup samples.

Several limitations warrant acknowledgment. First, the retrospective design of this study inherently carries risks of selection bias. A particular limitation in our model development is the relatively low number of events per variable (EPV). Although the EPV in our study met the minimum recommended threshold of 5, it did not reach the more widely accepted threshold of 10. A relatively low EPV increases the risk of overfitting and may result in model estimates that are overly dependent on the specific dataset, especially considering the very-high hazard ratios observed (e.g., HR of 28.1 for LNS ≥ 10 mm) and the unusually high C-index in the external validation set (0.95, 95% CI: 0.93–0.98). To mitigate these risks, we performed 1000 bootstrap resampling, including the variable selection step (Table S11), and conducted subgroup analyses (Table S12). Both consistently identified TMR and LNS as key predictors, providing additional evidence of model robustness. Nevertheless, the relatively small sample size and limited number of events suggest that our findings should be interpreted as exploratory and require confirmation in larger, more diverse cohorts. Second, there was a notable imbalance in the involvement of pathological lymph nodes between the development cohort (1% positive) and the validation cohort (7% positive). This discrepancy likely reflects the heterogeneity of lymph node dissection practices across different centers. Furthermore, it indicates that the validation cohort represents a relatively higher-risk population, which may potentially inflate the performance of the model. Future studies utilizing prospective, multicenter cohorts will be essential to further validate the robustness and generalizability of our findings. Third, although we categorized tumor margin irregularity (TMR) by percentage of circumference to improve standardization, it remains a subjective feature. Inter-reader agreement was excellent (kappa 0.85–0.87), but reproducibility among less experienced readers is uncertain, which may affect the reliability of the score in routine practice. Future research could explore more objective and quantitative approaches, such as radiomics-based metrics, to assess tumor margin irregularity. Fourth, the excretory phase was not included, which may have limited the assessment of collecting system invasion. Future studies should incorporate this phase for further validation. Finally, we used recurrence-free survival (RFS) as the primary endpoint, given the rarity of cancer-specific deaths in PRCC; this may limit generalizability to other survival outcomes.

## Conclusion

We developed a simple, practical preoperative radiological score based on tumor margin irregularity and regional lymph node size to predict recurrence in PRCC. Within our multicenter cohort, this score showed excellent predictive performance and may serve as a valuable adjunct to existing postoperative tools, helping to identify high-risk patients and guide individualized management. Given the retrospective design and potential for overfitting, prospective validation is essential before routine clinical adoption.

## Supplementary information

ELECTRONIC SUPPLEMENTARY MATERIAL

## Data Availability

The datasets generated and analyzed during the current study are not publicly available but are available from the corresponding author on reasonable request.

## Abbreviations

AUC: Area under the receiver operating characteristic curve
C-index: Concordance Index
CSS: Cancer-specific survival
CT: Computed tomography
ECOG-PS: Eastern Cooperative Oncology Group Performance Status
GRANT: GRade, Age, Nodes, and Tumor
ICC: Intraclass correlation coefficient
IQR: Interquartile range
ISUP: International Society of Urological Pathology
LNS: Lymph node size
PRCC: Papillary renal cell carcinoma
RCC: Renal cell carcinoma
RFS: Recurrence-free survival
SSIGN: Stage, Size, Grade, and Necrosis
TMR: Tumor margin regularity
VENUSS: VEnous tumor thrombus, NUclear grade, Size, T and N Stage
