# Supplementary material for: A preoperative CT-based radiological score for predicting recurrence in papillary renal cell carcinoma: a multicenter validation study
Source: Insights Imaging. 2025 Dec 8;16:272. doi: 10.1186/s13244-025-02161-9 (PMC12686295; doi:10.1186/s13244-025-02161-9)
Supplement: Supplementary file 1 — ELECTRONIC SUPPLEMENTARY MATERIAL [file 13244_2025_2161_MOESM1_ESM.pdf]

A Preoperative CT-Based Radiological Score for Predicting Recurrence in Papillary Renal Cell Carcinoma: A Multicenter Validation Study

ELECTRONIC SUPPLEMENTARY MATERIAL

Supplementary Material 1

Detailed Scoring and Grouping for Several Pathological Prognostic Models

The VENUSS score was calculated as 2 (venous tumour thrombus) + 2 (tumor size ＞4 cm) + 1 (pT2) + 2 (pT3 or pT4) + 3 (pN1) + 2 (Nuclear grade 3 or 4), and 0 otherwise. Patients with 0 to 2 scores are considered low risk, those with 3 to 5 scores are considered intermediate risk, and patients with 6 or more scores are considered high risk [1].

The SSIGN score was calculated as 2 (pT1b) + 3 (pT2) + 4 (pT3a) + 4 (pT3b, pT3c, and pT4) + 2 (pN1 and pN2) + 1 (tumor size ≥10 cm) + 1 (grade 3) + 3 (grade 4) + 1 (necrosis), and 0 otherwise. Patients with 0 to 2 scores are considered low risk, those with 3 to 5 scores are considered intermediate risk, and patients with 6 or more scores are considered high risk [2].

Leibovich et al. [3] model involved stratifying patients into low risk group (grades 1–2 without fat invasion and thrombus), intermediate risk group (grade 3 without fat invasion and thrombus), and high-risk group (grade 4 or with fat invasion or any thrombus level).

The GRANT score was calculated as 1 (Age≥ 60) + 1 (pT3b, pT3c, and pT4) + 1 (pN1 and pN2) + 1 (grade 3 and grade 4), and 0 otherwise. Patients with 0 to 1 scores are considered low risk, those with ≥2 scores are considered high risk [4].

Reference

1. Klatte T, Gallagher KM, Afferi L et al (2019) The VENUSS prognostic model to predict disease recurrence following surgery for non-metastatic papillary renal cell carcinoma: development and evaluation using the ASSURE prospective clinical trial cohort. BMC Med 17: 182.

2. Frank I, Blute ML, Cheville JC, Lohse CM, Weaver AL, Zincke H (2002) An outcome prediction model for patients with clear cell renal cell carcinoma treated with radical nephrectomy based on tumor stage, size, grade and necrosis: the SSIGN score. J Urol 168: 2395-2400.

3. Leibovich BC, Lohse CM, Cheville JC et al (2018) Predicting Oncologic Outcomes in Renal Cell Carcinoma After Surgery. Eur Urol 73: 772-780.

4. Buti S, Puligandla M, Bersanelli M et al (2018) Validation of a new prognostic model to easily predict outcome in renal cell carcinoma: the GRANT score applied to the ASSURE trial population. Ann Oncol 29: 1604.

Supplementary Material 2

Detailed Imaging Acquisition

Renal or abdominal enhanced CT scans were acquired from multiple hospitals using various CT parameters (Table S1). The general CT procedure consisted of first performing a routine unenhanced scan. This was followed by intravenous injection of a contrast agent (1.5–2 mL/kg) into an antecubital vein at an injection rate of 2.5–4 mL/s using a power injector. Two postcontrast phases were obtained the corticomedullary phase at 25–40 seconds after the start of injection and the nephrographic phase at 50–95 seconds after injection.

Supplementary Material 3

Detailed follow-up strategy

For patients with localized papillary renal cell carcinoma (PRCC), the general protocol is to perform abdominal and chest imaging twice a year for the first 3 years and once a year thereafter. For patients with locally advanced PRCC, every 3 months for the first year, twice yearly for the next 2 years, and then annually.

Supplementary Material 4

Statistical Analysis Details

For baseline parameters, continuous variables were presented as means with standard deviations if normally distributed, or as medians with interquartile ranges if non-normally distributed. Categorical variables were expressed as proportions. Group comparisons were conducted using the student's t-test or MannWhitney U test for continuous variables and the χ^2 test for categorical variables. Intraobserver agreement among three radiologists was investigated by Fleiss' kappa value for binary variables and the intraclass correlation coefficient (ICC) for continuous variables. Agreement was considered poor (kappa or ICC < 0.2), fair (kappa or ICC: 0.2–0.4), moderate (kappa or ICC: 0.4–0.6), substantial (kappa or ICC: 0.6–0.8), or excellent (kappa or ICC > 0.8).

Prior to constructing a multivariable model, the correlations (Pearson's correlation) between candidate variables was examined to check the collinearity. Correlations greater than 0.8 were considered potential collinearity among factors. These relationships were visualized using a correlation heatmap. Multicollinearity was also checked with variance inflation factor (VIF), which is well high the threshold of 10 indicating multicollinearity issues present.

Table S1. The detailed parameters of the CT examination.

	Total (n=266)	Development set (n=152)	Validation set (n=114)
Manufacturer			
SIEMENS	114 (43)	94 (62)	20 (18)
GE	38 (14)	25 (16)	13 (11)
UIH	24 (9)	21 (14)	3 (3)
TOSHIBA	13 (5)	12 (8)	1 (1)
Philips	77 (29)	0 (0)	77 (67)
Pixel size			
0.5-0.7mm	128 (48)	78 (51)	50 (44)
0.7-0.9mm	138 (52)	74 (49)	64 (56)
Slice thickness			
1-2.5mm	35 (13)	0 (0)	35 (31)
3mm	60 (23)	21 (14)	39 (34)
5mm	171 (64)	131 (86)	40 (35)
Tube voltage			
100 kVp	33 (12)	19 (13)	14 (12)
120 kVp	222 (84)	128 (84)	94 (83)
140 kVp	11 (4)	5 (3)	6 (5)

Note. —Values are numbers, with percentages in parentheses. kVp =peak kilovoltage.

Table S2. Definitions of the imaging predictors.

Imaging features	Definition and category
R.E.N.A.L score	The R.E.N.A.L. nephrometry score includes the following components: Radius, Exophytic or Endophytic tumor location, Nearness of the deepest tumor portion to the collecting system or sinus, Anterior or Posterior tumor location, and its Location relative to the polar line [1-2].
Radius (tumor size as maximal diameter)	The largest tumor diameter was measured on the transverse CT image during the nephrographic phase. The R subcategories were scored on a 1-, 2-, or 3-point scale as follows:<br>• Lesions ≤ 4 cm: 1 point<br>• Lesions > 4 cm but < 7 cm: 2 points<br>• Lesions ≥ 7 cm: 3 points
Exophytic or endophytic location of the tumor	The E subcategories were scored on a 1-, 2-, or 3-point scale as follows:<br>• Lesions projecting more than 50% outside the renal cortex: 1 point<br>• Lesions projecting less than 50% outside the renal cortex: 2 points<br>• Entirely endophytic lesions: 3 points
Nearness of the deepest portion of the tumor to the collecting system or sinus	The N subcategories were scored on a 1-, 2-, or 3-point scale as follows:<br>• Lesions ≥ 7 mm from the collecting system or renal sinus: 1 point<br>• Lesions > 4 mm but < 7 mm from the collecting system: 2 points<br>• Lesions ≤ 4 mm from the central collecting system: 3 points
Anterior or posterior location of the tumor	No points are assigned. Tumors located primarily on the ventral surface of the kidney are designated as "anterior (a)"; tumors on the dorsal renal surface are designated as "posterior (p)". Tumors that do not fit into these categories, such as purely lateral or central apical lesions, are assigned the designation "x"
Location relative to the polar line	The L subcategories were scored on a 1-, 2-, or 3-point scale as follows:<br>• Lesions entirely above or below the polar line: 1 point<br>• Lesions crossing the polar line: 2 points<br>• Lesions crossing more than 50% of the polar line, crossing the axial renal midline, or entirely between the polar lines: 3 points
Calcification	The presence of calcifications within the tumor [3] (Figure 2-A).
CT necrosis	The presence of necrosis was determined if ill-defined, hypodense areas of the tumor did not enhance during both the corticomedullary and nephrographic phases [4] (Figure 2-B).
Peritumoral Neovascularity	The presence of peritumoral vasculature [4] (Figure 2-C).
CT vein invasion	Venous invasion was defined as an intraluminal filling defect observed within a segmental (branch) or main renal vein on post-contrast imaging [5] (Figure 2-G).
CT collecting system invasion	Evidence of collecting system invasion, defined as a filling defect within the collecting system [6] (Figure 2-F).
CT renal sinus fat invasion	An ill-defined or irregular margin between the central tumor edge and any

	part of the renal sinus fat, or the presence of enhancing tumor tissue within the sinus fat, was noted [5] (Figure 2-D).
CT perinephric fat invasion	An ill-defined or irregular margin was observed between the peripheral tumor edge and the perinephric fat, or enhancing tumor tissue was seen within the perinephric fat [5] (Figure 2-E).
Tumor margin regularity (TMR)	Tumor margin regularity, as partly referenced in Tanaka's study [7], is classified into three categories:<br>• TMR 1: Completely regular (Figure 3 A-B)<br>• TMR 2: Irregular, with less than 50% of the entire circumference affected, including local micro-protrusions (Figure 3C) or obvious local protrusions (Figure 3D)<br>• TMR 3: Widely irregular, with 50% or more of the entire circumference affected, including widespread micro-protrusions (Figure 3E) or obvious widespread protrusions (Figure 3F)
Pattern of enhancement	The pattern of enhancement was classified into four types based on the proportion of cystic and solid components [8]:<br>• Homogeneous enhancement (Figure 2-H).<br>• Solid or predominantly solid lesions with small areas of low attenuation (Figure 2-I).<br>• Lesions with mixed solid and low-attenuation areas (Figure 2-J).<br>• Predominantly low-attenuation lesions with peripheral enhancement (Figure 2-K).
Regional lymph node size (LNS)	Regional lymph nodes were evaluated in three locations: hilar, side-specific (pre-/para-aortic or pre-/para-caval), and inter-aorto-caval. The largest lymph node in these areas was selected for assessment. According to Gershman's study [9], lymph nodes with a short-axis diameter of 7 mm are associated with a 20% predicted risk of lymph node involvement. Based on this, preoperative regional lymph node size (LNS) was classified using a three-point scale (Figure 3):<br>• LNS 1: No enlarged lymph nodes or a short-axis diameter < 7 mm (Figure 3G, H).<br>• LNS 2: Slightly enlarged lymph nodes, with a short-axis diameter ≥ 7 mm but < 10 mm (Figure 3I, J).<br>• LNS 3: Significantly enlarged lymph nodes, with a short-axis diameter ≥ 10 mm (Figure 3K, L).
Degree of enhancement	The attenuation values of renal tumors (TAV) and the renal cortex (CAV) were measured on the same axial slice of contrast-enhanced CT. Specifically, $TAV_{PCP/CMP/NP}$ and $CAV_{PCP/CMP/NP}$ represent the CT attenuation values of renal tumors and adjacent renal cortex in the pre-contrast phase (PCP), corticomedullary phase (CMP), and nephrographic phase (NP), respectively. The final TAV and CAV values were calculated as the average of two independent measurements performed by radiologists. To minimize individual variations in CT imaging

	due to differences in contrast agent metabolism, we derived the following metrics:<br>Net enhancement values:<br>TEV (tumor enhancement value) = $TAV_{CMP/NP} - TAV_{PCP}$<br>CEV (cortex enhancement value) = $CAV_{CMP/NP} - CAV_{PCP}$<br>Relative enhancement ratio (RER):<br>$RER_{CMP/NP} = TEV_{CMP/NP} \div CEV_{CMP/NP}$<br>The RER was used as an indicator of enhancement strength across phases. ROI selection was performed by three radiologists independently, following these guidelines:<br>（1）ROIs for renal tumors and normal renal cortex were consistent in size and location across the three phases of contrast-enhanced CT.<br>（2）Circular or oval ROIs were drawn to encompass the most uniform and maximally enhanced solid areas of the renal tumor while avoiding regions of necrosis, calcification, vasculature, or cystic components.<br>（3）Each ROI was measured twice, and the average value was used for the final analysis.<br>Examples of ROIs used for measuring TAV and CAV are provided in Figure 2 L–N.

Reference

1. Kutikov A, Uzzo RG (2009) The R.E.N.A.L. nephrometry score: a comprehensive standardized system for quantitating renal tumor size, location and depth. J Urol 182: 844-853.

2. Parsons RB, Canter D, Kutikov A, Uzzo RG (2012) RENAL nephrometry scoring system: the radiologist's perspective. AJR Am J Roentgenol 199: W355-359.

3. Li C, Cen D, Liu Z, Liang C (2018) Presence of Intratumoral Calcifications and Vasculature Is Associated With Poor Overall Survival in Clear Cell Renal Cell Carcinoma. J Comput Assist Tomogr 42: 418-422.

4. Hötker AM, Karlo CA, Zheng J et al (2016) Clear Cell Renal Cell Carcinoma: Associations Between CT Features and Patient Survival. AJR Am J Roentgenol 206: 1023-1030.

5. Sokhi HK, Mok WY, Patel U (2015) Stage T3a renal cell carcinoma: staging accuracy of CT for sinus fat, perinephric fat or renal vein invasion. Br J Radiol 88: 20140504.

6. Karlo CA, Kou L, Di Paolo PL et al (2016) Renal cell carcinoma: A nomogram for the CT imaging-inclusive prediction of indolent, non-clear cell renal cortical tumours. Eur J Cancer 59: 57-64.

7. Tanaka H, Fukuda S, Kimura K et al (2023) Defining Tumour Shape Irregularity for Preoperative Risk Stratification of Clinically Localised Renal Cell Carcinoma. Eur Urol Open Sci 48: 36-43.

8. Zhang J, Lefkowitz RA, Ishill NM et al (2007) Solid renal cortical tumors: differentiation with CT. Radiology 244: 494-504.

9. Gershman B, Takahashi N, Moreira DM et al (2016) Radiographic size of retroperitoneal lymph nodes predicts pathological nodal involvement for patients with renal cell carcinoma: development of a risk prediction model. BJU Int 118: 742-749.

Table S3: Univariable and multivariable cox proportional hazard analyses for postoperative recurrence-free survival in development set.

	Univariable Cox Proportional Analysis			Multivariable Cox Proportional Analysis			
	Regression Coefficient	Hazard Ratio	P Value	Regression Coefficient	Hazard Ratio	P Value	Score
**R.E.N.A.L score**							
Radius (tumor size as maximal diameter) (cm)							
≤ 4	Ref.	Ref.	Ref.	...	...	...	...
> 4 but < 7	0.48	1.61 (0.51-5.08)	.42	...	...	...	...
≥ 7	1.22	3.40 (0.88-13.2)	.08	...	...	...	...
Exophytic or endophytic location of the tumor							
≥ 50%	Ref.	Ref.	Ref.	...	...	...	...
< 50%	0.84	2.31 (0.78-6.89)	.13	...	...	...	...
Endophytic	0.5	1.65 (0.33-8.19)	.54	...	...	...	...
Nearness of the deepest portion of the tumor to the collecting system or sinus (mm)							
≥ 7	Ref.	Ref.	Ref.	...	...	...	...
> 4 but < 7	-17.3	0 (0-Inf)	.99	...	...	...	...
≤ 4	1.29	3.61 (0.82-16)	.09	...	...	...	
**Qualitative and quantitative CT features**							
CT necrosis	0.45	1.57 (0.21-11.94)	.66	...	...	...	...
CT renal sinus fat invasion	0.96	2.62 (0.93-7.36)	.06	...	...	...	...
CT perinephric fat invasion	1.38	3.96 (0.89-17.57)	.07	...	...	...	...
RER_{cmp} (%)	0.18	1.19 (0.09-15.58)	0.89				
RER_{np} (%)	-0.67	0.51 (0.02-16.57)	0.71				

Table S4. the multicollinearity analysis of significant factors after univariable cox proportional analysis in traning set.

Factors	VIF value	Tolerance
Location relative to the polar line	1.13	0.88
Calcification	1.14	0.88
Pattern of enhancement	1.02	0.98
Tumor margin regularity	2.10	0.48
Peritumoral neovascularity	1.36	0.74
CT renal vein invasion	1.47	0.68
CT collecting system invasion	1.67	0.60
Regional lymph node size	1.36	0.74

Note. —VIF = variance inflation factor.

Table S5. Interobserver agreement of CT features in PRCC.

Imaging Features	Frequency			Kappa value or ICC*	Agreement
	R1	R2	R3		
Radius (tumor size as maximal diameter) (cm)					
≤4	155 (58)	158 (60)	156 (59)	0.90	Excellent
> 4 but < 7	79 (30)	80 (30)	79 (30)		
≥7	32 (12)	28 (10)	31 (11)		
Exophytic or endophytic location of the tumor					
≥50%	147 (55)	129 (48)	150 (57)	0.75	Substantial
<50%	87 (33)	98 (37)	83 (31)		
Endophytic	32 (12)	39 (15)	33 (12)		
Nearness of the deepest portion of the tumor to the collecting system or sinus (mm)					
≥7	78 (29)	86 (32)	77 (29)	0.80	Substantial
> 4 but < 7	26 (10)	25 (10)	27 (10)		
≤4	162 (61)	155 (58)	162 (61)		
Location relative to the polar line					
Entirely above or below the polar line	155 (58)	132 (50)	154 (58)	0.69	Substantial
Cross the polar line	80 (31)	53 (20)	80 (30)		
>50% cross the polar line crosses the axial renal midline or entirely between the polar lines	31 (11)	81 (30)	32 (12)		
CT necrosis (present vs absent)	16 (6)	15 (6)	19 (7)	0.85	Excellent
Calcification (present vs absent)	29 (11)	40 (15)	30 (11)	0.86	Excellent
Pattern of enhancement					
Homogeneous enhancement	108 (41)	110 (41)	110 (41)	0.86	Excellent
Solid or predominantly solid lesions with small areas of low attenuation	79 (30)	74 (28)	77 (29)		
Lesions with mixed solid and low-attenuation areas	19 (7)	26 (10)	19 (7)		
Predominantly low-attenuation lesions with peripheral enhancement	60 (23)	56 (21)	60 (23)		

Peritumoral neovascularity (present vs absent)	12 (5)	8 (3)	10 (4)	0.86	Excellent
CT collecting system invasion (present vs absent)	20 (8)	22 (8)	20 (8)	0.83	Excellent
CT renal vein invasion (present vs absent)	6 (2)	5 (2)	6 (2)	0.94	Excellent
CT renal sinus fat invasion (present vs absent)	61 (23)	61 (23)	61 (23)	0.53	Moderate
CT perinephric fat invasion (present vs absent)	10 (4)	8 (3)	11 (4)	0.43	Moderate
RERcmp (%)[†]	0.23±0.21	0.22±0.23	0.27±0.18	0.87[*]	Excellent
RERnp (%)[†]	0.30±0.19	0.30±0.20	0.30±0.18	0.84[*]	Excellent

Unless stated otherwise, data in parentheses are percentages. [†]Data are mean±SD; R1, R2, and R3 were reviewers with 7, 10, and 5 years of experiences in urological imaging, respectively. RERcmp = Relative enhancement ratio of corticomedullary phase，RERnp = Relative enhancement ratio of nephrographic phase. Agreement was considered poor (κ or ICC <0.2), fair (κ or ICC: 0.2–0.4), moderate (κ or ICC: 0.4–0.6), substantial (κ or ICC: 0.6–0.8), or excellent (κ or ICC >0.8). [*]Interobserver agreement was investigated by computing the Fleiss'κ value for ordinal/categorical variables and the intraclass correlation coefficient (ICC) for continuous variables.

Table S6. Univariate cox regression analysis of the radiological score and clinicopathological factors with RFS in two set.

	Development set (n=156)		External test set (n=114)	
	HR (95% CI)	P value	HR (95% CI)	P value
Sex (female vs male)	2.64 (0.96-7.30)	0.06	0.98 (0.27-3.56)	0.97
Age	0.97 (0.93-1.01)	0.12	1.01 (0.96-1.07)	0.64
ECOG-PS (1-4 vs 0)	0.74 (0.21-2.63)	0.64	4.70 (1.28-17.30)	0.02
Tumor size	1.22 (1.03-1.45)	0.02	1.42 (1.25-1.62)	<0.001
Surgical method (PN vs RN)	0.34 (0.11-1.06)	0.06	0.19 (0.05-0.68)	0.01
Lymphadenectomy (Yes vs No)	3.96 (0.52-30.18)	0.18	67.98 (16.24-284.5)	<0.001
Adjuvant treatment (Yes vs No)	23.46 (8.02-68.68)	<0.001	25.37 (7.35-87.55)	<0.001
ISUP Grade (grade 3-4 vs 1-2)	3.85 (1.40-10.62)	0.009	6.39 (2.06-19.87)	0.001
Necrosis (present vs absent)	15.5 (4.26-56.45)	<0.001	7.31(2.23-23.96)	0.001
Sarcomatoid Differentiation (present vs absent)	8.53 (1.11-65.50)	0.04	NA (NA-NA)	NA
TNM stage (III vs II vs I)	3.87 (2.26-6.64)	<0.001	8.56 (3.88-18.86)	<0.001
Radiological score	5.61 (3.50-8.99)	<0.001	6.67 (3.48-12.80)	<0.001

Note. —RN = Radical nephrectomy，PN = Partial nephrectomy, ISUP = International Society of Urological Pathology, TNM = Tumor Node Metastasis, HR = Hazard Ratio.

Table S7. Multivariate Cox regression analysis of the radiological score and clinicopathological factors with RFS in two set.

	Training set (n=152)		Independent validation set (n=114)	
	HR (95% CI)	P value	HR (95% CI)	P value
Sex (female vs male)	0.79 (0.14-4.47)	0.79	7.24 (0.35-150.6)	0.20
Age	0.97 (0.91-1.04)	0.45	1.07 (0.91-1.25)	0.44
ECOG-PS (1-4 vs 0)	0.41 (0.04-4.60)	0.47	0.11 (0.004-2.46)	0.16
Tumor size	0.91 (0.63-1.31)	0.61	1.28 (0.76-2.15)	0.36
Surgical method (PN vs RN)	0.58 (0.08-4.42)	0.60	10.81 (0.61-192.6)	0.11
Lymphadenectomy (Yes vs No)	0.18 (0.01-2.77)	0.14	18.29 (1.35-248.0)	0.03
Adjuvant treatment (Yes vs No)	2.45 (0.51-11.78)	0.22	0.1 (0.001-14.15)	0.36
ISUP Grade (grade 3-4 vs 1-2)	0.21 (0.02-2.02)	0.18	4.35 (0.51-37.42)	0.18
Necrosis (present vs absent)	4.17 (0.24-73.83)	0.33	1.137 (0.018-71.84)	0.95
Sarcomatoid Differentiation (present vs absent)	6.06 (0.10-355.2)	0.39	NA (NA-NA)	NA
TNM stage (III vs II vs I)	1.64 (0.57-4.75)	0.36	13.11 (1.34-128.6)	0.02
Radiological score (high-score vs low-score)	118.8 (6.96-2027)	0.001	76.69 (3.82-1539)	0.005

Note. —RN = Radical nephrectomy，PN = Partial nephrectomy, ISUP = International Society of Urological Pathology, TNM = Tumor Node Metastasis, HR = Hazard Ratio.

Table S8. The Recurence-free, cancer-specific, and overall survival outcome of PRCC based on the radiological score.

Variables	Total (n=266)	Development set (n=152)			Validation set (n=114)		
		Low risk patients (n=140)	High risk patients (n= 12)	P value	Low risk patients (n=98)	High risk patients (n= 16)	P value
RFS,month†	51.7 (27.5;77)	69.8 (43.8;112.7)	32.5 (8.0;47.0)	0.002	34.3 (25.0;59.8)	19.6 (12.0;31.9)	0.002
Recurence (%)	28(10.5)	4 (2.9)	11 (91.7)	<0.001	1 (1)	12 (75)	<0.001
1-year RFS rate, %	96.2	98.6	66.7	<0.001	100	75	<0.001
3-year RFS rate, %	92.5	97.1	50	<0.001	99	43.8	<0.001
5-year RFS rate, %	89.9	97.1	16.7	<0.001	99	25	<0.001
CSS,month†	54.9 (30.1;79)	69.8 (44.2;113.0)	57.2 (28.1;82.4)	0.207	34.3 (25.0;59.8)	30 (23.9;48.8)	0.533
Died of pRCC (%)	18 (6.8)	3 (2.1)	7 (58.3)	<0.001	1 (1)	7 (43.8)	<0.001
1-year CSS rate, %	100	100	100	NA	100	100	NA
3-year CSS rate, %	95.9	98.6	66.7	<0.001	99	75	0.001
5-year CSS rate, %	94	97.9	58.3	<0.001	99	56.3	<0.001
OS,month†	54.9 (30.1;79)	70.2 (44.9;113.8)	44.9 (28.3;82.4)	0.053	34.3 (25.0;59.8)	30.0 (23.9;48.8)	0.533
Died of all cause	25 (9.4)	4(2.9)	11(91.7)	<0.001	2 (2)	8 (50)	<0.001
1-year OS rate, %	100	100	100	NA	100	100	NA
3-year OS rate, %	94.7	98.6	50	<0.001	98	75	<0.001
5-year OS rate, %	92.1	97.9	33.3	<0.001	98	50	<0.001

Note. —Data are numerators and/or data in parentheses are percentages. †Data are medians; data in parentheses are IQRs. Patients were stratified into either a high-risk (2-3 points) or a low-risk group (0-1 points) according to the radiological score. RFS = Recurence-Free Survival, CSS = Cancer-Specific Survival, OS = Overall Survival.

Table S9. Comparison of prognostic performance of different models in small PRCC (≤ 3cm) from all sets (n=104).

	Performmance		
Model	C-index	95% CI	P value
Radiological score	0.80	0.53-1.08	-
VENUSS group	0.67	0.40-0.94	0.35
2018 leibovich group	0.67	0.36-0.97	0.28
SSIGN group	0.67	0.40-0.94	0.35
GRANT group	0.64	0.37-0.92	0.21

Note. —VENUSS = VEnous tumour thrombus, NUclear grade, Size, T and N Stage, GRANT = GRade, Age, Nodes, and Tumor, SSING = Stage, Size, Grade and Necrosis. P values were computed by comparing with the radiological score.

Table S10. The numbers of patients in different subgroups.

	subgroups	total number	number of recurrences	P值
VENUSS (N=48)	low-risk radiological score with VENUSS intermedium-high-risk	43	3	0.0045
	high-risk radiological score with VENUSS low-risk	5	3	
SSIGN (N=42)	low-risk radiological score with SSIGN intermedium-high-risk	37	2	0.0021
	high-risk radiological score with SSIGN low-risk	5	3	
Leibovich (N=61)	low-risk radiological score with Leibovich intermedium-high-risk	55	2	<0.0001
	high-risk radiological score with Leibovich low-risk	6	5	
GRANT (N=41)	low-risk radiological score with GRANT high-risk	26	1	<0.0001
	high-risk radiological score with GRANT low-risk	15	13	

Table S11. Frequency of variable selection across 1000 bootstrap resamples.

Variable	Selection frequency (%)
Regional lymph node size (LNS)	93.1
Tumor margin regularity (TMR)	84.9
Calcification	65.3
Peritumoral neovascularity	50.7
CT renal vein invasion	46.0
CT collecting system invasion	41.3
Location relative to the polar line	31.3
Pattern of enhancement	19.8

Table S12. The performance of radiological model in different subgroups.

		Performance		1000 bootstrap	
	Subgroup	C-Index	95% CI	C-index	95% CI
Age	≤ 60 (n=150)	0.918	0.847-0.989	0.918	0.845-0.988
	> 60 (n=116)	0.901	0.783-1.019	0.901	0.776-1.028
Sex	Male (n=201)	0.917	0.85-0.984	0.917	0.848-0.985
	Female (n=65)	0.905	0.782-1.028	0.905	0.774- 1.027
Tumor size	<4cm (n=153)	0.874	0.684-1.064	0.874	0.673- 1.071
	>4cm (n=113)	0.898	0.835-0.961	0.897	0.832- 0.961
ISUP grade	Low-grade (n=200)	0.874	0.752-0.996	0.873	0.745- 0.998
	High-grade(n=66)	0.904	0.851-0.957	0.904	0.847- 0.958
T stage	T1 (n=218)	0.904	0.782-1.026	0.904	0.775-1.024
	T2-3 (n=48)	0.838	0.746-0.93	0.838	0.741-0.934

Table S13. Comparison of model performance between the original continuous linear model and the integer-based score model

original continuous linear	Set	C-index	P value
	Train set	0.876 (0.770- 0.982)	-
	Validation set	0.962 (0.933- 0.990)	-
the integer-based score	Train set	0.878 (0.772-0.983)	0.763
	Validation set	0.954 (0.927- 0.982)	0.189

Supplementary Figures

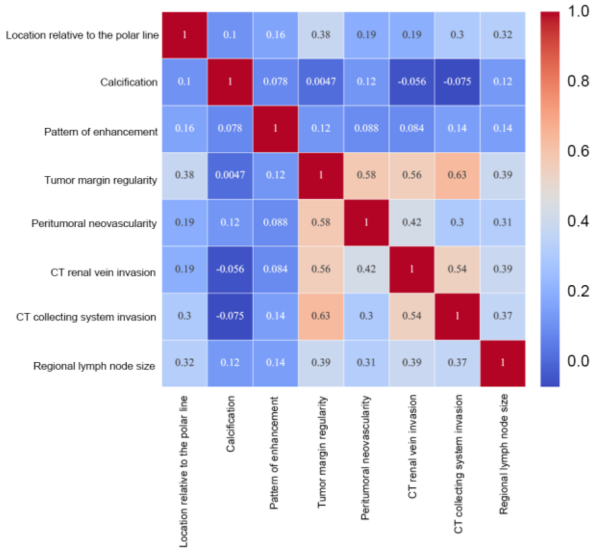

Supplementary Figure S1. The heatmap illustrates the collinearity among various parameters.

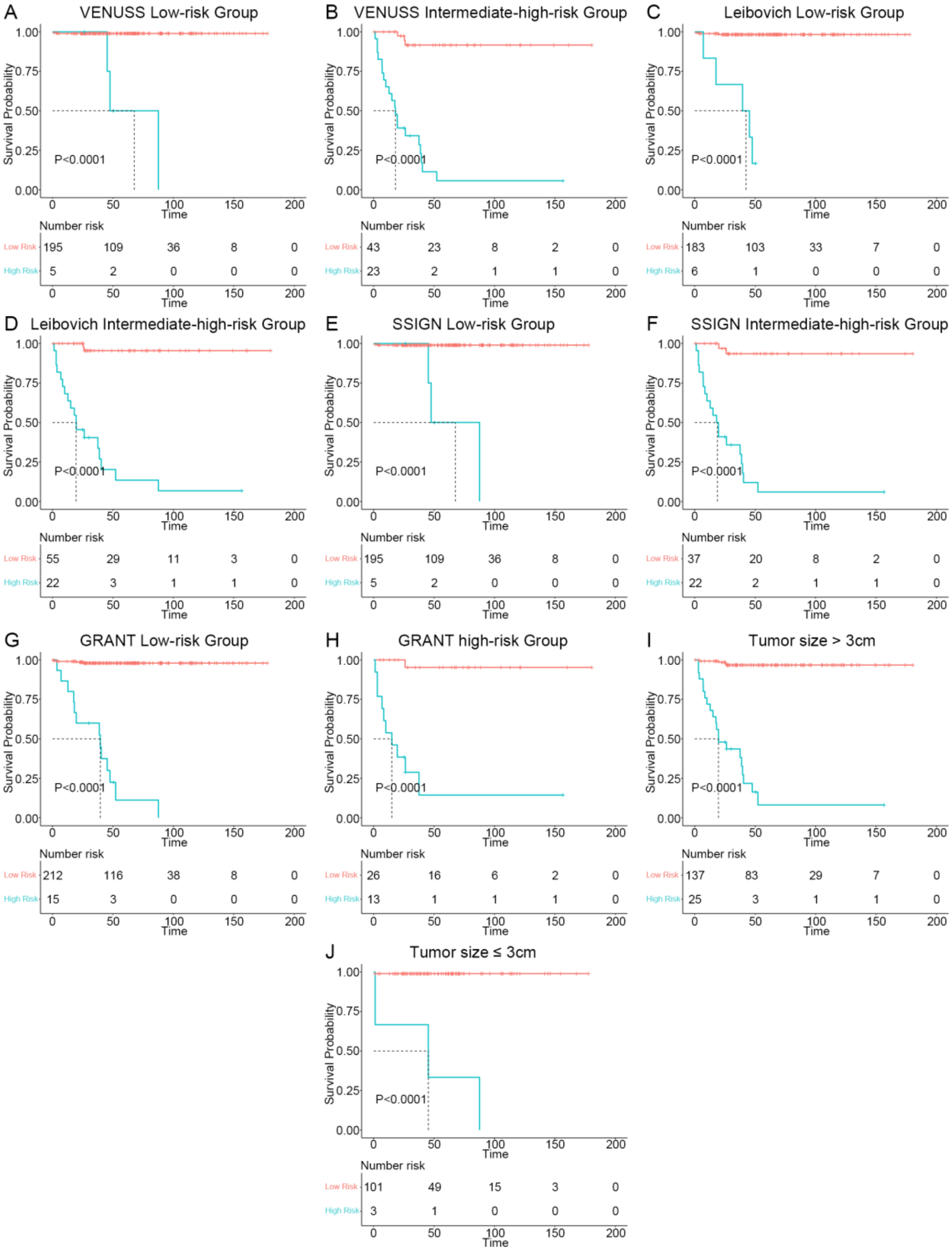

Supplementary Figure S2. Kaplan-Meier survival analysis for RFS of the radiological scoring system in different subgroups stratified by clinicopathological risk factors. VENUSS group (A, B), 2018 Leibovich group (C, D), SSIGN group (E, F), GRANT group (G, H), and Tumor Size (I, J).

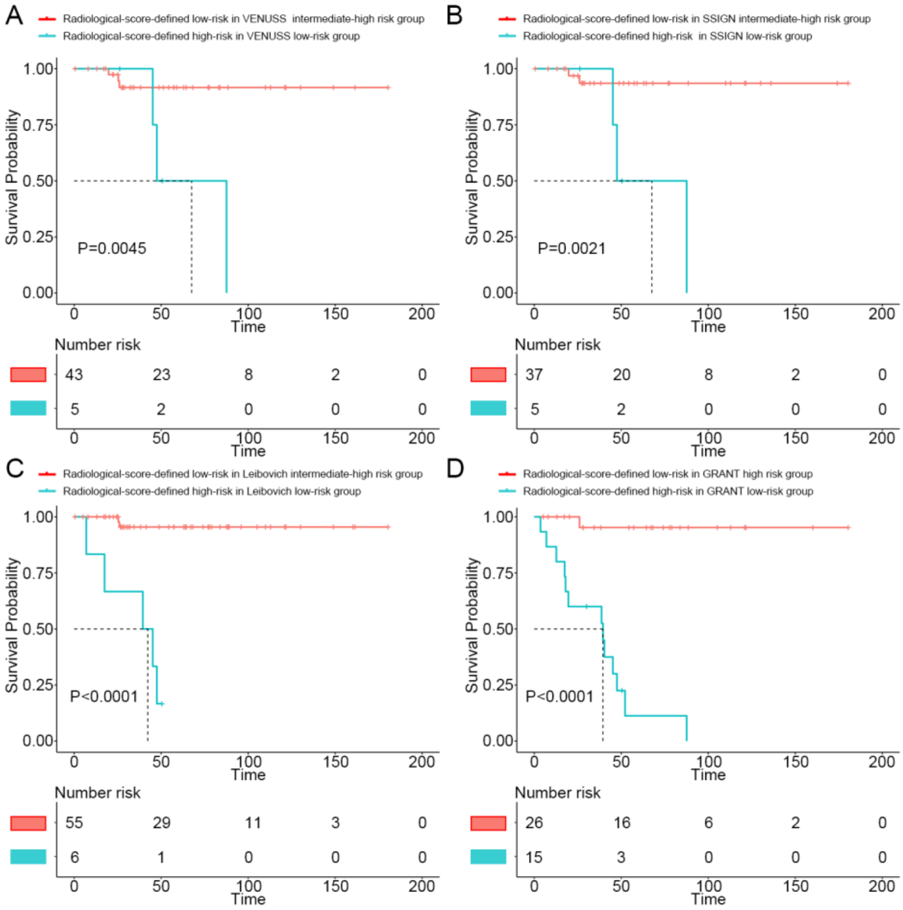

Supplementary Figure S3. Kaplan–Meier survival analysis in radiological risk-score-defined low-risk and high-risk patients stratified by pathological prognostic model. Kaplan-Meier survival analysis for RFS of the low-risk radiological score with VENUSS intermedium-high-risk group patients and high-risk radiological score with VENUSS low-risk group patients (A), low-risk radiological score with SSIGN intermedium-high-risk group patients and high-risk radiological score with SSIGN low-risk group patients (B), low-risk radiological score with Leibovich intermedium-high-risk group patients and high-risk radiological score with Leibovich low-risk group patients (C), low-risk radiological score with GRANT high-risk group patients and high-risk radiological score with GRANT low-risk group patients (D).
